# 

**Published:** 1855-04

**Authors:** 


APRIL 1855.

THE

### Dental Register of the West.

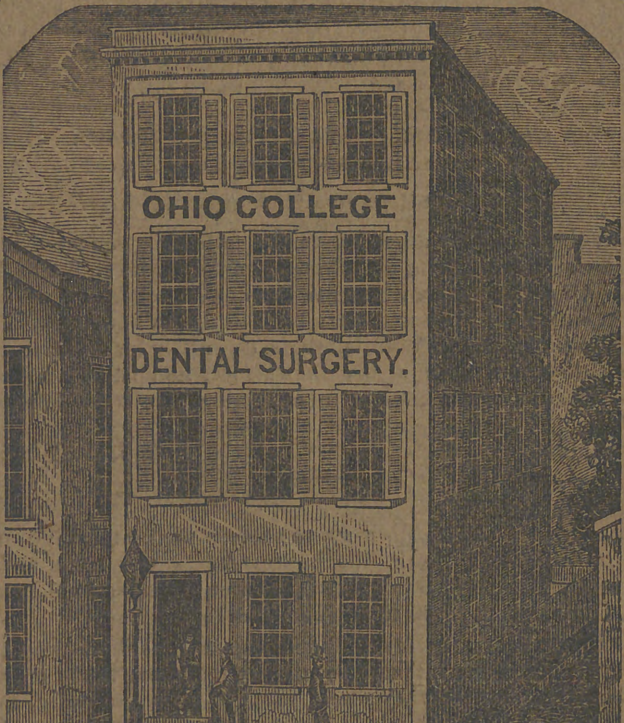

JAMES TAYLOR, M. D., D. D. S., Editor.

Published Quarterly at $2 per Annum.

CINCINNATI:
T. WRIGHTSON & CO., PRINTERS,
167 Walnut Street.

